# Natural infection of *Aedes albopictus* with the *w*AlbB strain and *Ae. aegypti* with the *w*Pip strain of *Wolbachia* in Iran

**DOI:** 10.1038/s41598-026-40993-7

**Published:** 2026-02-21

**Authors:** Fateh Karimian, Sara Rahimy, Hemn Yousefi, Mostafa Salehi-Vaziri, Abbasali Raz, Mohammad Hassan Pouriayevali, Eslam Moradi-Asl, Naseh Maleki-Ravasan

**Affiliations:** 1 Department of Parasitology, Pasteur Institute of Iran, Tehran, Iran; 2 Malaria and Vector Research Group, Biotechnology Research Center, Pasteur Institute of Iran, Tehran, Iran; 3 Department of Arboviruses and Viral Hemorrhagic Fevers (National Reference Laboratory), Pasteur Institute of Iran, Tehran, Iran; 4 Arthropod-Borne Diseases Research Center, Ardabil University of Medical Sciences, Ardabil, Iran

**Keywords:** *Wolbachia* symbionts, Invasive mosquitoes, Emerging infectious diseases, Arbovirus control, Cytoplasmic incompatibility, Iran, Diseases, Ecology, Ecology, Microbiology, Zoology

## Abstract

The global resurgence of *Aedes*-borne arboviruses (dengue, chikungunya, and Zika) underscores the need for innovative vector control strategies. In Iran—a region at risk for arbovirus emergence due to invasive *Aedes albopictus* and *Ae. aegypti* mosquitoes— we investigated the natural occurrence of *Wolbachia* infections. Screening of field-collected and laboratory-reared mosquitoes revealed the presence of two *Wolbachia* strains: *w*AlbB in *Ae. albopictus* and *w*Pip in *Ae. aegypti*. Notably, detection of *w*Pip in the wild *Ae. aegypti* represents a potential first report globally, as this species has been historically considered uninfected in natural populations. *Wolbachia* prevalence was significantly higher in laboratory-reared mosquitoes and *Ae. albopictus*. Multivariable analysis confirmed that *Ae. aegypti* had significantly lower odds of *Wolbachia* infection compared to *Ae. albopictus* (adjusted OR = 0.12, 95% CI: 0.03–0.45, *p* = 0.002), and infection odds were also significantly lower in field-collected mosquitoes from Guilan (OR = 0.28), Mazandaran (OR = 0.05), and Hormozgan (OR = 0.14) provinces relative to insectary-reared specimens (all *p* < 0.05). These findings provide critical baseline data for evaluating the feasibility of *Wolbachia*-based interventions in Iran, where invasive *Aedes* populations could facilitate arbovirus transmission. This study highlights the importance of characterizing native *Wolbachia* infections to inform future biocontrol strategies against emerging mosquito-borne diseases.

**Supplementary Information:**

The online version contains supplementary material available at 10.1038/s41598-026-40993-7.

## Introduction

Invasive species are non-native organisms that cause environmental, economic, or public health harm^1,2^. Among them, *Aedes* (*Stegomyia*) *aegypti* (Linnaeus, 1762) and *Aedes* (*Stegomyia*) *albopictus* (Skuse, 1895), stand out as globally invasive vectors of arboviruses such as dengue, chikungunya, and Zika^3^. Their spread is facilitated by global trade and travel, and their establishment in new regions—including Iran—poses increasing public health risks.

A day-biting *Ae. albopictus*, commonly known as the Asian tiger mosquito, is highly invasive and capable of transmitting at least 22 arboviruses, including dengue, chikungunya, Zika, yellow fever, Japanese encephalitis, West Nile, Rift Valley fever and Sindbis viruses^4,5^. It thrives in tropical to temperate climates and can survive colder winters, enabling establishment in regions inhospitable to other mosquitoes^5^. *Ae. aegypti*, an anthropophilic and endophilic species, is similarly adapted to urban environments in tropical climates and transmits multiple arboviruses^6^. Their co-occurrence increases disease transmission risk, complicates vector control, and may lead to interspecific interactions such as competition or hybridization^7–9^.

Controlling invasive *Aedes* mosquitoes presents numerous challenges, including behavioral plasticity, insecticide resistance, difficulties in surveillance, and the impacts of human behaviors related to urbanization, global travel and trade, climate change, and economic constraints^6,10–13^. The development of innovative and effective tools, such as *Wolbachia*-based interventions could help to mitigate disease transmission and suppress mosquito populations^14^.

*Wolbachia pipientis* is the most ubiquitous and maternally transmitted bacterium that naturally infects certain nematodes and various insect species, including mosquitoes. Although approximately 30% of mosquitoes are naturally infected with *Wolbachia*, wild *Ae. aegypti* mosquitoes are predominantly not infected^15^. However, *Wolbachia* strains such as *w*Mel, *w*AlbB, and *w*MelPop have successfully been introduced into *Ae. aegypti* in numerous trials for biological control purposes^16^. In contrast, *Ae. albopictus* is naturally infected with either low-density (*w*AlbA) or high-density (*w*AlbB) *Wolbachia* strains, or both^17^. Compared to *w*AlbA, *w*AlbB significantly enhances cytoplasmic incompatibility (CI), reduces the transmission of arboviruses, and effectively interferes with viral replication in mosquitoes^18,19^. Co-infection with both *w*AlbA and *w*AlbB provides a balance between host fitness and pathogen-blocking capabilities, increasing the resilience of *Wolbachia* to environmental pressures and ensuring stable transmission across generations^20^.

This study was designed to address the information gap regarding the status of *Wolbachia* infection in invasive *Aedes* populations in Iran. To achieve this goal, we screened the prevalence and diversity of *Wolbachia* in field-collected and lab-reared invasive mosquitoes, specifically *Ae. albopictus* and *Ae. aegypti*, from different origins and biotopes in Northern and Southern Iran using *wsp*-based nested PCR analysis. These findings will facilitate a better understanding of the *Wolbachia*-virus-*Aedes* interaction and establish a platform for *Wolbachia*-based control strategies for invasive mosquitoes.

## Methods

### Ethical statement

The present research was performed in harmony with the relevant recommendations and regulations of the Ethics Committee of the Pasteur Institute of Iran, Tehran, Iran (ethical codes: IR.PII.REC.1402.043 and IR.PII.REC.1400.083). The experiment procedure was reviewed and approved by the aforesaid committee and did not require any additional approval.

### Origin of mosquitoes and specimen collection

**Fig. 1:**
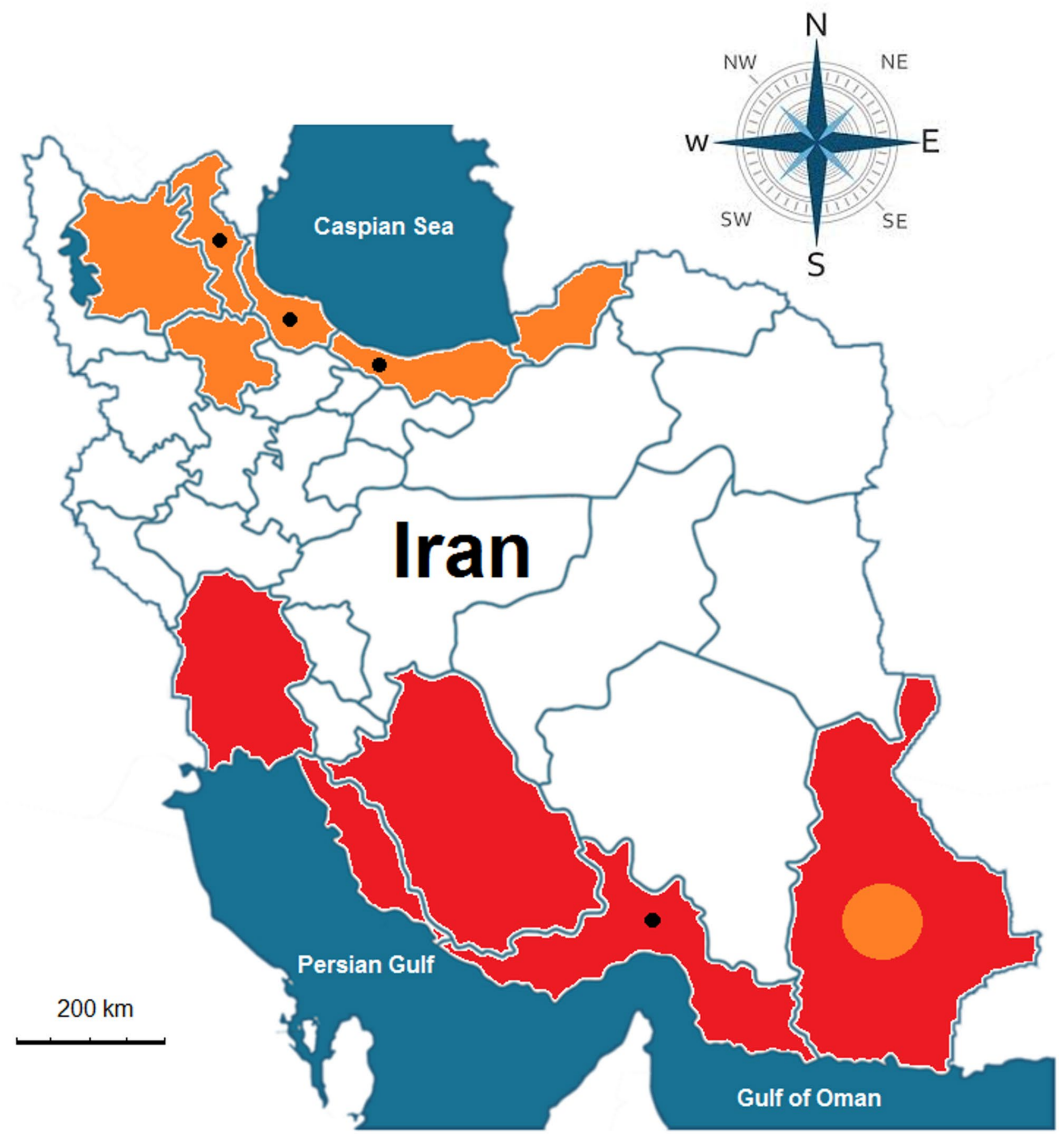
Geographic distribution of invasive *Aedes* mosquitoes in Iran. Black dots indicate collection sites for *Aedes albopictus* and *Ae. aegypti*. Shaded regions show areas where establishment of these species has been documented^52^. *Ae. albopictus* (orange) is established along the coastal provinces of the Caspian Sea, while *Ae. aegypti* (red) is established in southern provinces bordering the Persian Gulf and the Sea of Oman (scale bar: 200 km).

This study investigated natural *Wolbachia* infections in invasive *Aedes* mosquitoes using specimens from both laboratory-reared and field-collected populations. Laboratory specimens of *Ae. aegypti* (originally from Southeast Asia) and *Ae. albopictus* (originally from Sistan and Baluchestan Province) were obtained from the National Insectary of Iran, where they were reared at a mean temperature of 26 °C ± 2 °C, 70 ± 10% relative humidity, and under 12:12 light and dark conditions^21^. Field-collected *Ae. albopictus* specimens were sampled from six counties across three northern provinces of Iran: Bileh Savar (Ardabil Province), Bandar Anzali, Khomam, and Shaft (Guilan Province), and Ramsar and Tonekabon (Mazandaran Province). The *Ae. aegypti* specimens were collected from three neighborhoods in Bandar Abbas City, Hormozgan Province (Fig. 1, Supplementary Table 1).

Adult mosquitoes from all locations were collected using manual mouth aspirators. Physiological status of abdomens (i.e. unfed or blood fed, semi-gravid or gravid) was determined via visual examination for female specimens where possible. Specimens were immediately transferred into individual 1.5 mL Eppendorf tubes and stored at −70 °C until morphological identification.

At each sampling site, geographic coordinates (latitude and longitude in decimal degrees) were recorded using a handheld GPS device (Garmin eTrex 30x) at the approximate center of the collection locality. Monthly mean temperature (°C) and relative humidity (%) corresponding to the sampling period were obtained from the nearest active meteorological stations operated by the Iran Meteorological Organization (2020–2023). Data were extracted as monthly averages specific to the collection months at each site.

Mosquitoes were morphologically identified to species level using a validated taxonomic key^22^ prior to molecular analysis.

### DNA extraction and detection of *Wolbachia* infection

The genomic DNA of adult mosquitoes from various origins and habitats in Northern and Southern Iran was extracted using the Collins method^23^. *Wolbachia* infection in mosquitoes was assessed using a nested PCR protocol based on the procedures and primers described in the literature^24,25^. In brief, PCR was conducted in a total volume of 25 µL, which included 2–5 µL of DNA extract (~ 0.2 µg), 12.5 µL of Taq 2× Green PCR Master Mix (Eurogenome GMbH, Germani), 1 µL of each primer (10 mM), and 5.5–8.5 µL of sterile water. The PCR was performed at 95 °C for 5 min (pre-heating), followed by 35 cycles of denaturation at 94 °C for 1 min, annealing at 55 °C for 1 min, and extension at 72 °C for 1 min, followed by a final extension at 72 °C for 7 min. Accordingly, 632 bp and 501 bp of partial *wsp* gene sequences were amplified for nest-1 and nest-2, respectively. The nest-1 PCR product was electrophoresed on a 1.5% agarose gel, while the nest-2 reaction was performed only for negative specimens.

To minimize contamination risks inherent to nested PCR, strict spatial and procedural separation was maintained: pre-PCR (DNA extraction, master mix preparation) and post-PCR (product analysis) steps were conducted in dedicated, physically isolated laboratories with unidirectional workflow. Each PCR run included negative controls (nuclease-free water) and positive controls (*Wolbachia*-infected *Phlebotomus papatasi*). All positive amplifications (*n* = 3) were confirmed via independent replicate PCRs. Contamination control measures included the use of aerosol-resistant filter tips, routine UV irradiation of work surfaces, and adherence to a documented contamination prevention protocol.

Successful amplicons were purified and sequenced using the same primers by Pishgam Company, Tehran, Iran.

### Sequence analysis and phylogenetic tree reconstruction

The obtained *Wolbachia* sequences were analyzed to assess infection prevalence, evaluate genetic diversity, and classify isolates into *Wolbachia* sub- and supergroups. To explore the genetic structure of *Wolbachia* populations in invasive mosquitoes in Iran, sequences from this study were analyzed both independently and in combination with homologous reference sequences retrieved from GenBank. After confirming sequence quality, multiple sequence alignments and phylogenetic analyses were performed using MEGA X software (version 10.2.6)^26^.

Two maximum likelihood (ML) phylogenetic analyses were conducted: (1) to determine the placement of our *Wolbachia* isolates among sequences from other *Aedes* species, and (2) to assess their phylogenetic position relative to established subgroups defined by Zhou, et al.^24^. The best-fit nucleotide substitution model for our *wsp* dataset — HKY + I+G — was selected using the built-in model selection tool in MEGA X based on the Bayesian Information Criterion (BIC). Phylogenetic trees were reconstructed under this model with 1000 ultrafast bootstrap replicates to assess node support. This approach significantly enhances the robustness and biological realism of our phylogenetic inference. Additionally, nucleotide sequences were translated into their corresponding amino acid sequences using the EMBOSS *Transeq* tool^27^. The sequences obtained in this study were deposited in the GenBank database.

### MLST-based strain typing

To complement the *wsp*-based classification and provide higher-resolution strain characterization, multi-locus sequence typing (MLST) was performed following the standardized *Wolbachia* MLST scheme^28^. Three housekeeping genes—*ftsZ*, *gatB*, and *groEL*—were analyzed. Reference sequences for these loci were retrieved from GenBank for *Wolbachia* strains infecting *Aedes* and related arthropods. Sequences were analyzed using MEGA software with the same defaults and settings as described in previous section. MLST profiles were compared with the PubMLST Wolbachia database (https://pubmlst.org/organisms/wolbachia-spp) to assign sequence types.

### Statistical analysis

To identify factors associated with *Wolbachia* infection status in mosquitoes, we performed multivariable logistic regression with infection (positive/negative) as the binary dependent variable. Independent variables included: mosquito species (*Ae. albopictus* vs. *Ae. aegypti*), origin (field-collected vs. laboratory-reared), sex (male vs. female), and collection site (categorical variable with insectary as the reference group). Model selection was performed using a backward stepwise approach based on the Akaike Information Criterion (AIC), retaining only variables with *p* < 0.05 in the final model. Odds ratios (OR) with 95% confidence intervals (CI) were calculated to quantify the strength and direction of associations.

Descriptive statistics (frequencies and percentages) were used to summarize *Wolbachia* prevalence across categories. All statistical analyses were conducted in *R* software (version 4.3.2) using the glm function with binomial family and logit link. Statistical significance was defined as *p* < 0.05.

## Results

### Species and gender composition of mosquitoes

**Fig. 2:**
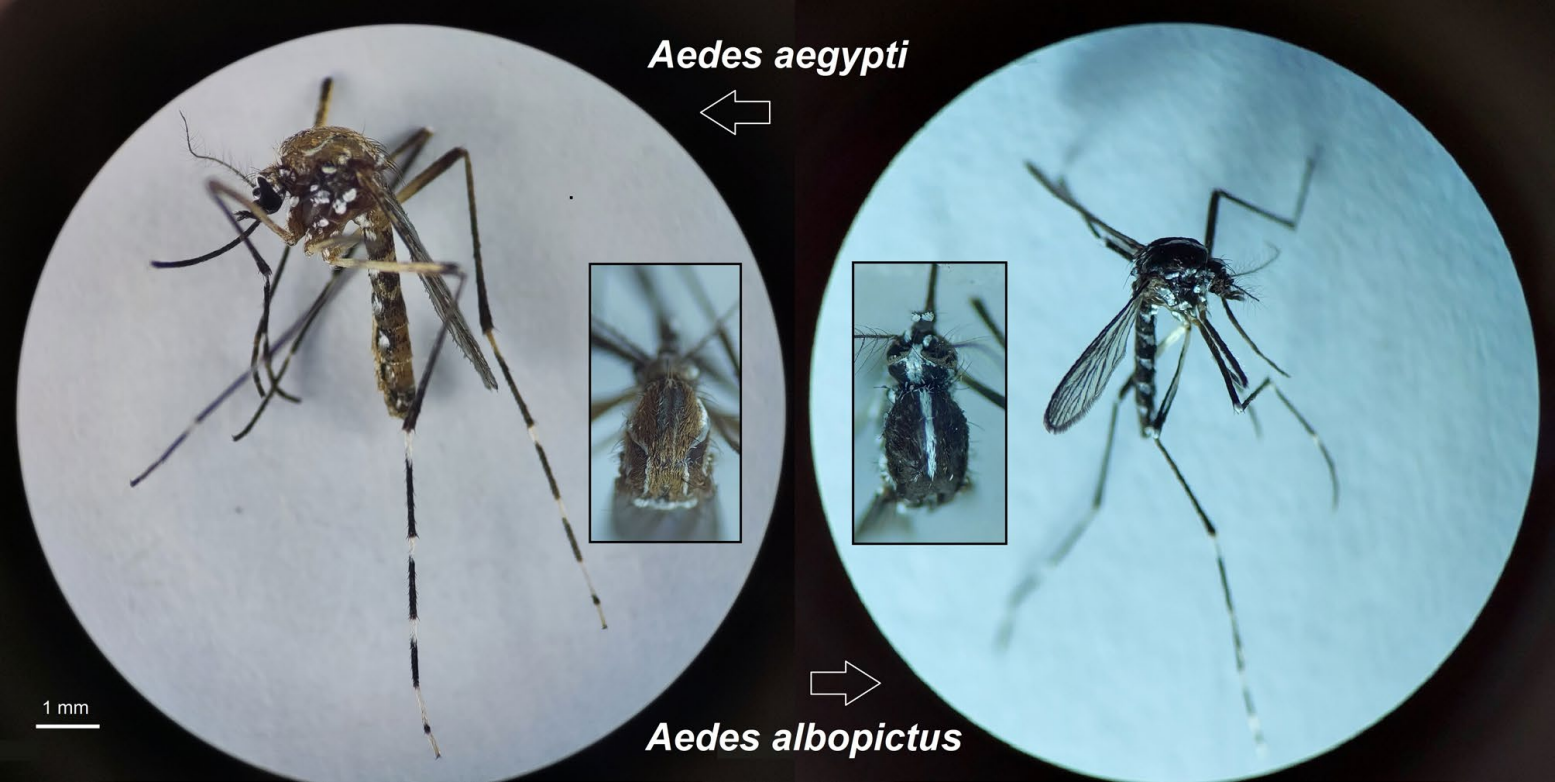
Diagnostic morphological features of *Aedes aegypti* (left; collected in Bandar Abbas City) and *Aedes albopictus* (right; reared at the National Insectary of Iran). Both species exhibit white banding (stripes) on the legs and scutum. Key distinguishing characters; *Ae. aegypti*: Silvery-white, lyre-shaped pattern on the dorsal scutum, *Ae. albopictus*: Single, median, longitudinal silvery-white stripe extending from the anterior to posterior margin of the scutum (scale bar: 0.1 mm, magnification: ×40).

A total of 777 mosquitoes, including 140 males and 637 females, were used in this study to investigate *Wolbachia* infection. The specimens belonged to two taxonomic species: *Ae. albopictus* (Skuse, 1894) with a sample size of 401, and *Ae. aegypti* (Linnaeus in Hasselquist, 1762) with a sample size of 376. Additionally, 228 mosquito specimens originated from an insectary, while 549 were collected from the fields. Notably, male specimens of *Ae. albopictus* were not captured from the fields of the study areas (Figs. 1 and 2; Table 1). The diagnostic characters of the two invasive *Aedes* spp. were so prominent that their morphological identification was performed with great precision. Only in two mosquito specimens from Bandar Abbas City, where scales and other diagnostic characters were lost, molecular identification with the barcode gene confirmed the identity of the suspected samples as *Ae. aegypti*.

**Table 1:** Determination of infection rates and *Wolbachia* subgroups of *Aedes albopictus* and *Ae. aegypti* mosquitoes from different sources in Iran.

Aedes species	Origin	Males tested/infected (%)	Females Tested/infected (%)	Physiological status		Wolbachia subgroups
				Fed	Unfed	
*Aedes albopictus*	Insectary	50/12 (24)	58/9 (15.5)	7	2	*w*AlbB
	Guilan Province	-	228/16 (7)	12	4	*w*AlbB
	Mazandaran Province	-	59/00 (0)	-	-	*w*AlbB
	Ardabil	-	6/1 (16.6)	0	1	*w*AlbB
*Aedes aegypti*	Insectary	50/0 (0)	70/0 (0)	-	-	-
	Hormozgan Province	40/2 (5)	216/6 (2.31)	5	1	*w*Pip
Total		140/14 (10)	637/32 (5)	24	8	*w*AlbB/*w*Pip

### *Wolbachia* detection and infection prevalence in mosquitoes

**Fig. 3:**
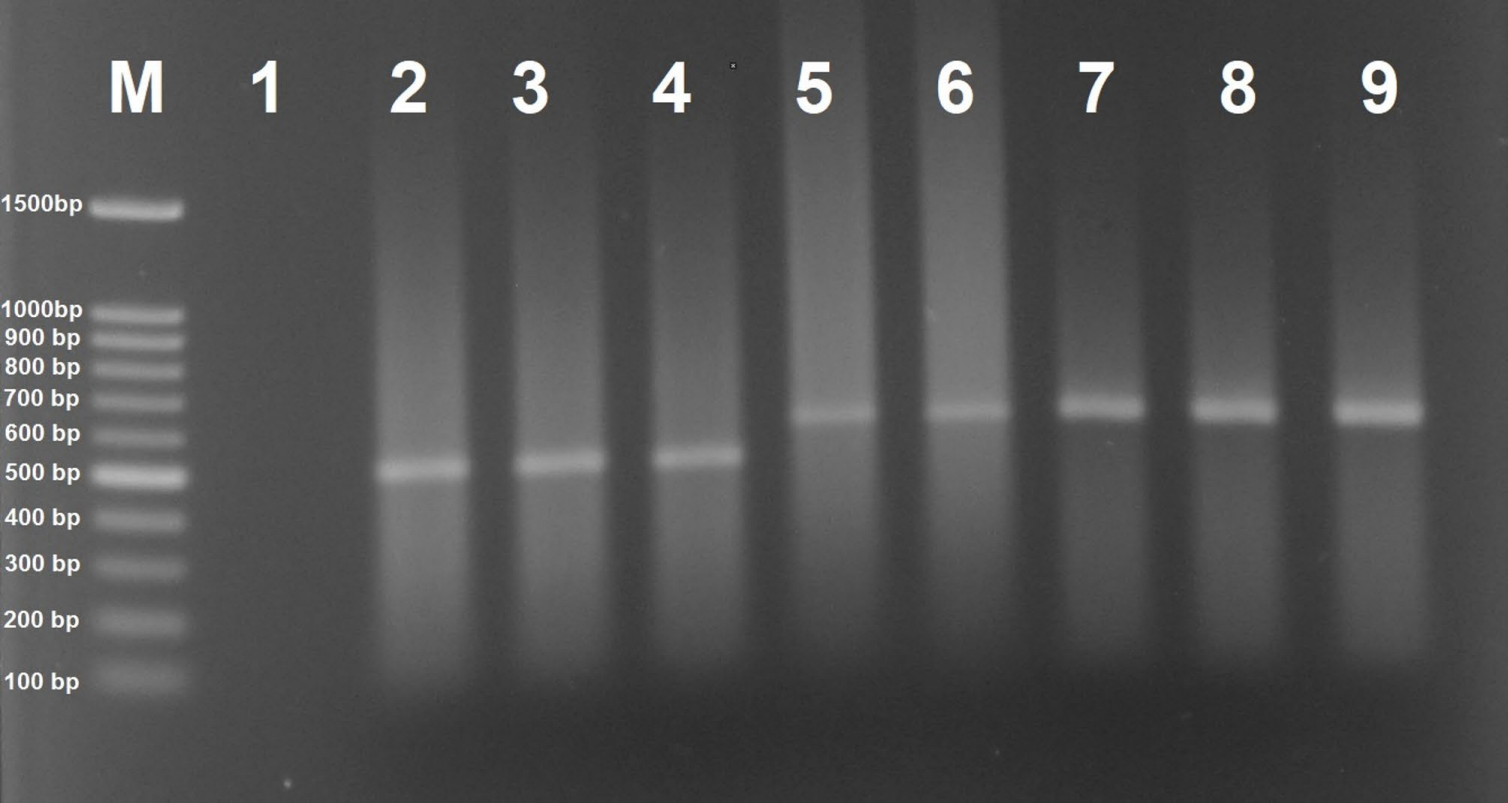
Species-specific nested PCR amplification of the *Wolbachia surface protein* gene (*wsp*) from *Aedes albopictus* and *Ae. aegypti* mosquitoes. Lanes: M, 100-bp ladder (Fermentas, USA); 1, negative control (nuclease-free water); 2, positive control (*w*Pap strain from *Phlebotomus papatasi*); 3–4, nest-2 PCR products (~ 500 bp); 5–9, nest-1 PCR products (~ 650 bp).

The nested PCR assay targeting the *wsp* gene successfully detected *Wolbachia* infections in field- and laboratory-collected invasive *Aedes* mosquitoes. Amplicons from the first and second rounds of nested PCR were approximately 650 bp and 500 bp in length, respectively (Fig. 3). The investigation of *Wolbachia* infection in invasive mosquitoes in Iran revealed interesting findings. Multivariable logistic regression identified mosquito species and collection site as significant predictors of *Wolbachia* infection (*p* < 0.05; Supplementary Table 2). Specifically, *Ae. aegypti* had significantly lower odds of infection compared to *Ae. albopictus* (OR = 0.12, 95% CI: 0.03–0.45, *p* = 0.002). Compared to insectary as the reference category, infection odds were significantly lower in Guilan (OR = 0.28, *p* = 0.01), Mazandaran (OR = 0.05, *p* = 0.03), and Hormozgan (OR = 0.14, *p* = 0.009). Variables including origin (lab vs. field) and sex (male vs. female) were not retained in the final model due to non-significance (both *p* > 0.05).

Among laboratory-reared *Ae. albopictus*, infection prevalence was 24% (12/50) in males and 15% (9/58) in females. In field-collected females, prevalence varied across sites, ranging from 0% to 17%. No *Wolbachia* infection was detected in laboratory-reared *Ae. aegypti* (males or females). In contrast, field-collected *Ae. aegypti* from Hormozgan Province showed infection rates of 5% (2/40) in males and 2% (6/216) in females. Overall, *Wolbachia wsp* gene sequences were amplified in 6% (46/777, 95% CI: 4.5–8.1) of all mosquitoes tested, regardless of species, sex, or origin (Table 1).

### Sequence analysis

Among 46 positive *Wolbachia* specimens, 17 isolates were randomly selected from various regions and species and sequenced successfully. Analyses of multiple sequence alignments revealed the presence of two distinct lineages of *Wolbachia* in the *Ae. albopictus* and *Ae. aegypti* populations. Given the 100% similarity among the samples, one sequence from males and one from females from each of the two *Wolbachia* groups were used for bioinformatics analyses. The BLAST results of the *wsp* gene sequences indicated that the *Wolbachia* isolates infecting *Ae. albopictus* were 100% identical to those found in *Ae. albopictus* samples from Spain (MT569498), China (MW718008), Philippines (OM304378), Panama (MH392336), Malaysia (MH418461), and USA (CP031221). Additionally, these *Wolbachia* isolates were identical to those found in *Ae. aegypti* samples from India (MF999246) and Malaysia (MN893358). Analysis of the *wsp* gene sequences from *Ae. aegypti* showed their dissimilarity to any *Wolbachia* detected in *Aedes* species. Instead, they were 100% identical to *Wolbachia* isolates found in *Culex quinquefasciatus* (OR455010) and *Culex pipiens* (OP292211) mosquitoes, as well as in lepidopterans such as *Falcaria lacertinaria* (OZ034799) and *Eurema mandarina* (AP028952). The sequences of the two *Wolbachia* groups have been deposited in the GenBank with accession numbers PV122193-PV122209.

### Allocations of *Wolbachia* isolates into sub/supergroup

**Fig. 4:**
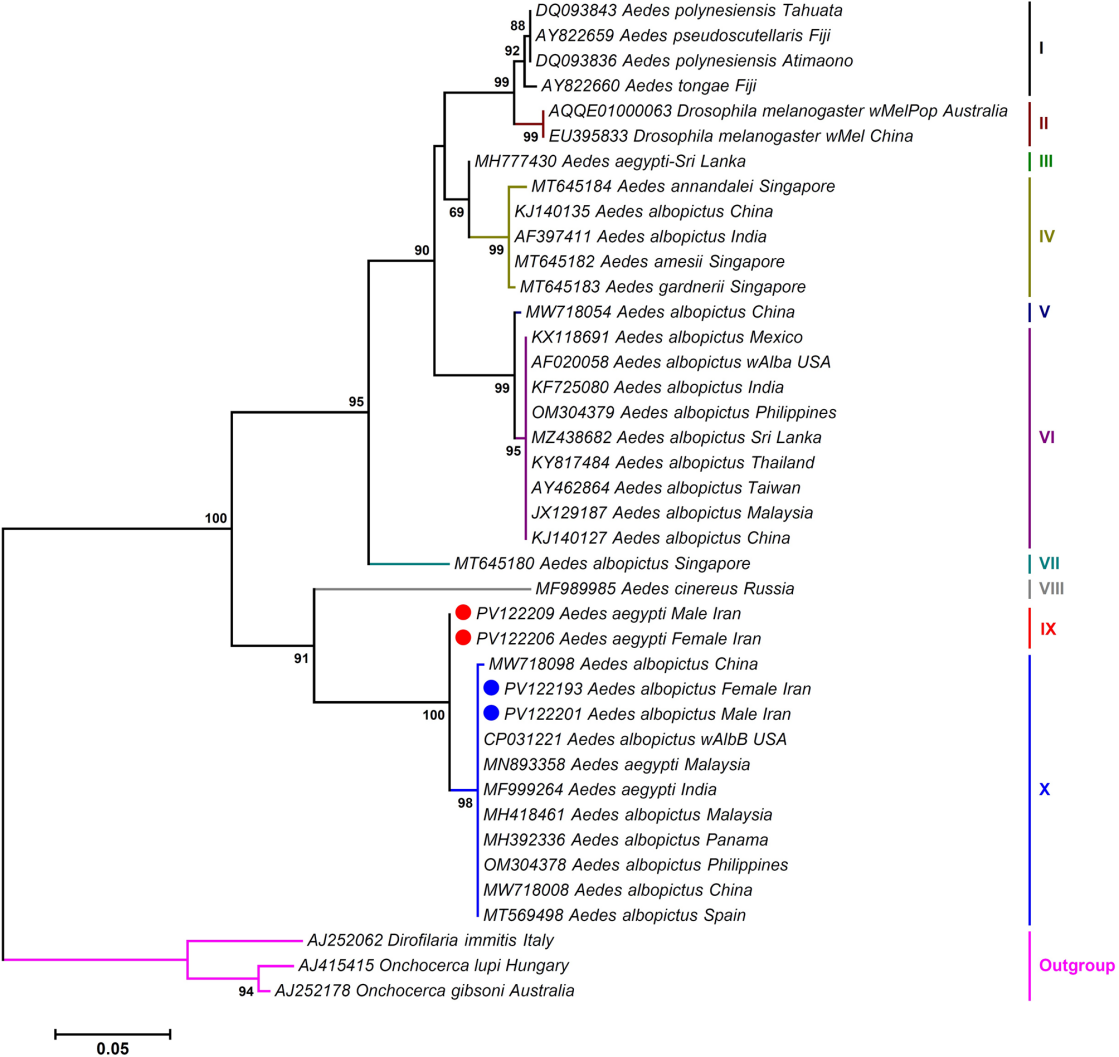
Maximum likelihood phylogenetic tree based on a 514-bp fragment of the *Wolbachia wsp* gene, showing the evolutionary placement of *Wolbachia* strains isolated from *Aedes aegypti* (red circles) and *Ae. albopictus* (blue circles) in this study among related strains from other *Aedes* species (GenBank-derived sequences). *Wolbachia* sequences from *Dirofilaria immitis* (AJ252062), *Onchocerca lupi* (AJ415415), and *Onchocerca gibsoni* (AJ252178) were used as outgroups to root the tree. Bootstrap support values (1,000 replicates) are shown at nodes; values < 50% are not displayed. The scale bar represents the number of nucleotide substitutions per site.

**Fig. 5:**
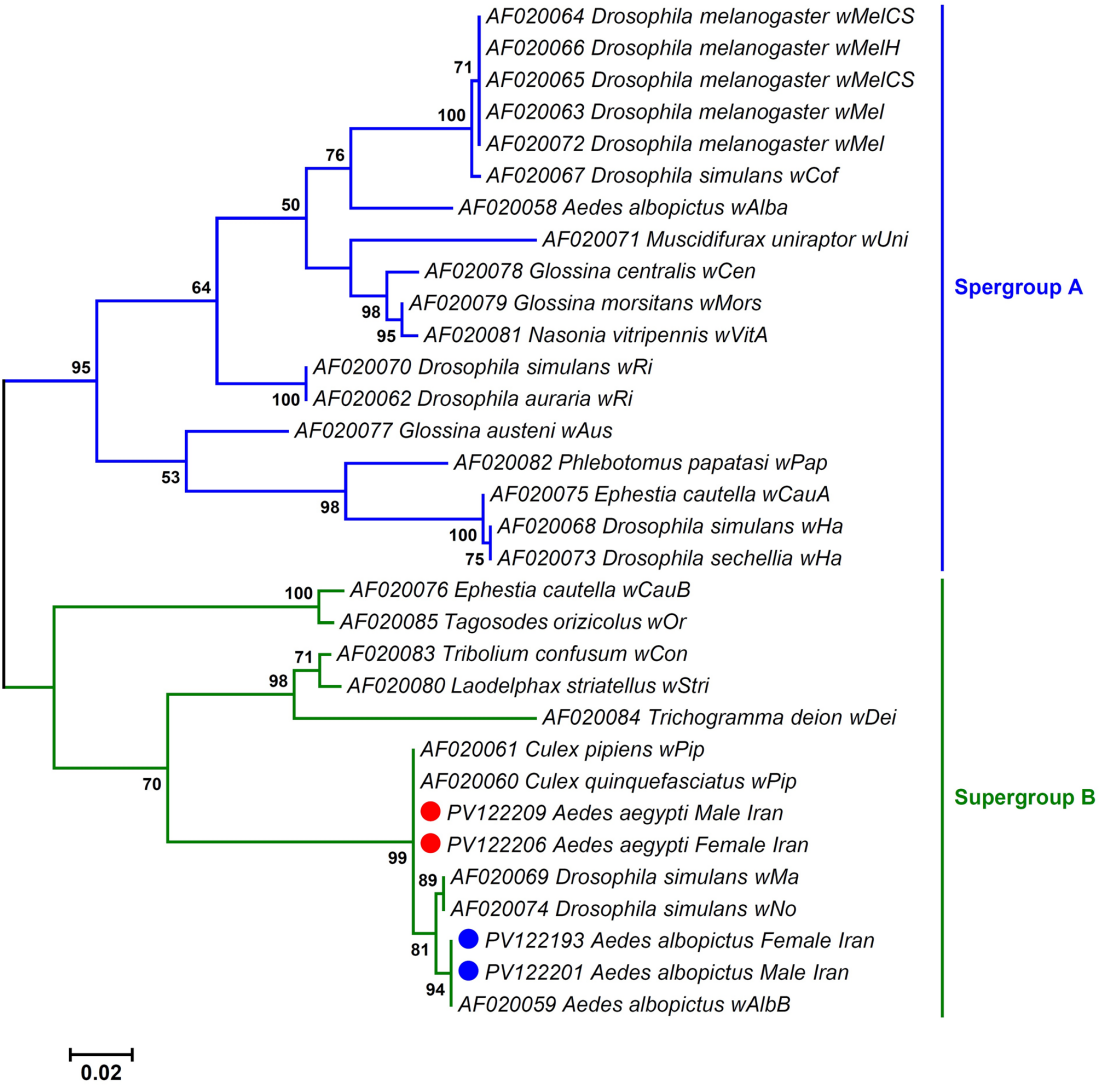
Maximum likelihood phylogenetic tree based on a 498-bp fragment of the *Wolbachia wsp* gene, illustrating the placement of *Wolbachia* strains from this study within the established *wsp*-based supergroup and subgroup classification system of Zhou, et al.^24^. Isolates from *Ae. aegypti* and *Ae. albopictus* in this study are marked with red and blue circles, respectively. Bootstrap values lower than 50% were omitted from the branches. The bar indicates substitutions per site.

**Fig. 6:**
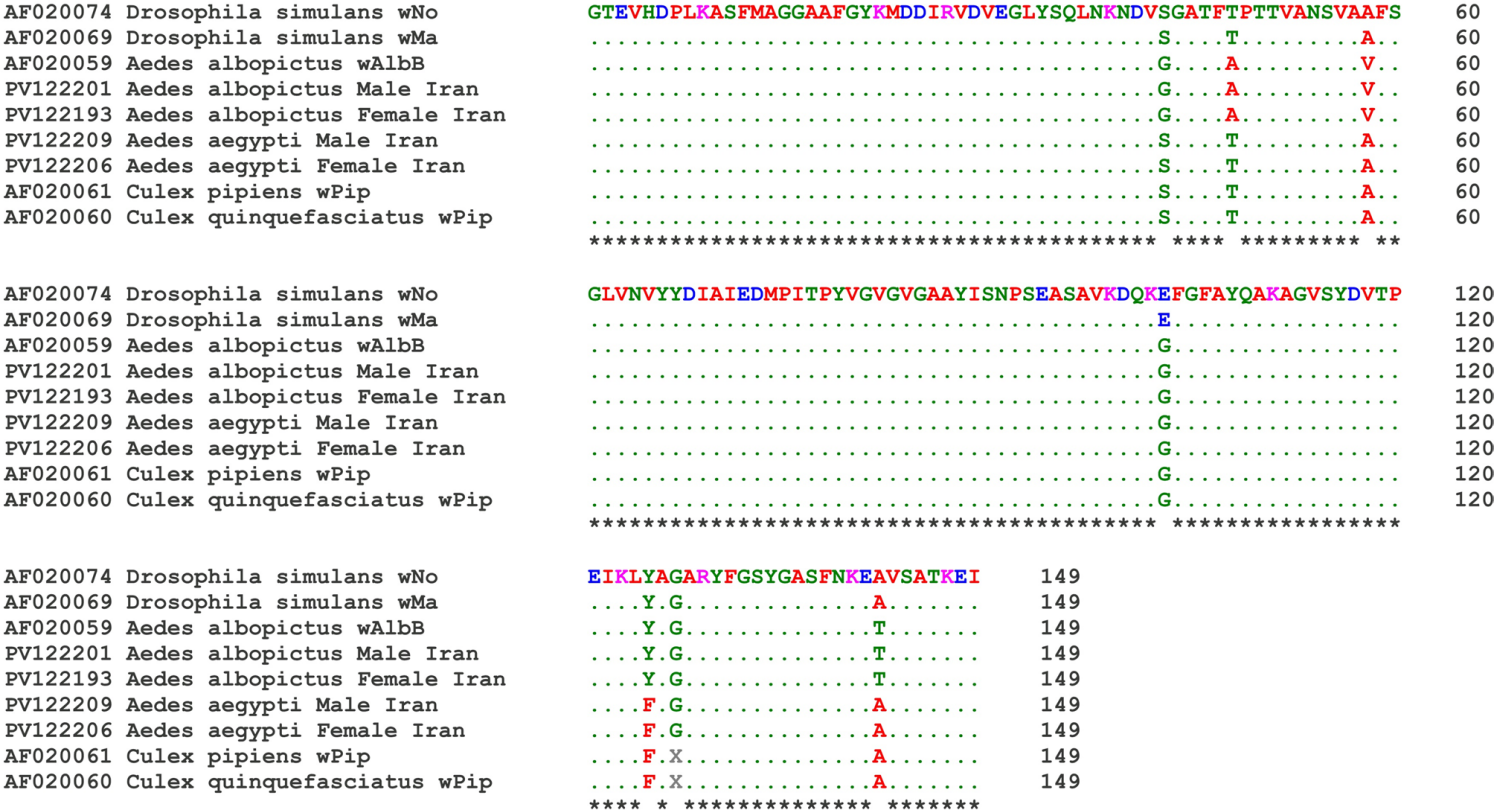
Amino acid sequence alignment of the *Wolbachia* surface protein (WSP) based on 149 deduced residues from four isolates obtained in this study (PV122193, PV122201, PV122206, PV122209; labeled “Iran”) and five closely related reference sequences (GenBank: AF020059, AF020060, AF020061, AF020069, AF020074). All sequences cluster within supergroup B in the phylogenetic tree (Fig. 5). The top sequence (AF020074) serves as the reference; dots (•) indicate identical amino acid residues. Dashes (–) represent gaps introduced to optimize alignment. Sequence differences highlight polymorphic sites potentially relevant to strain typing or functional divergence.

Phylogenetic analysis based on *wsp* gene sequences revealed that all *Wolbachia* isolates identified in this study belonged to supergroup B, strongly supported by the monophyly of lineages and high bootstrap values. The initial ML phylogenetic tree illustrated the placement of *Wolbachia* isolated from *Ae. albopictus* within the branch corresponding to *Wolbachia* sequences from both *Ae. albopictus* and *Ae. aegypti* retrieved from the GenBank (BS = 98%; Fig. 4). However, the *Wolbachia* sequences from *Ae. aegypti* clustered separately and independently from other *Aedes Wolbachia* isolates (BS = 100%; Fig. 4). The second tree further indicated that *Wolbachia* was isolated from *Ae. albopictus* and *Ae. aegypti* corresponded to the *Wolbachia* subgroups *w*AlbB (BS = 94%) and *w*Pip (BS = 99%), respectively (Fig. 5). Alignment of the deduced amino acid sequences revealed five (S43G, T48A, A58V, E102G, and A142T) mutations in the *w*AlbB subgroup and three (E102G, Y125F, and G127X) mutations in the *w*Pip subgroup of *Wolbachia* when compared to the sequence of no strain (AF020074; Fig. 6).

### MLST-based strain characterization of *wPip* lineage

**Fig. 7:**
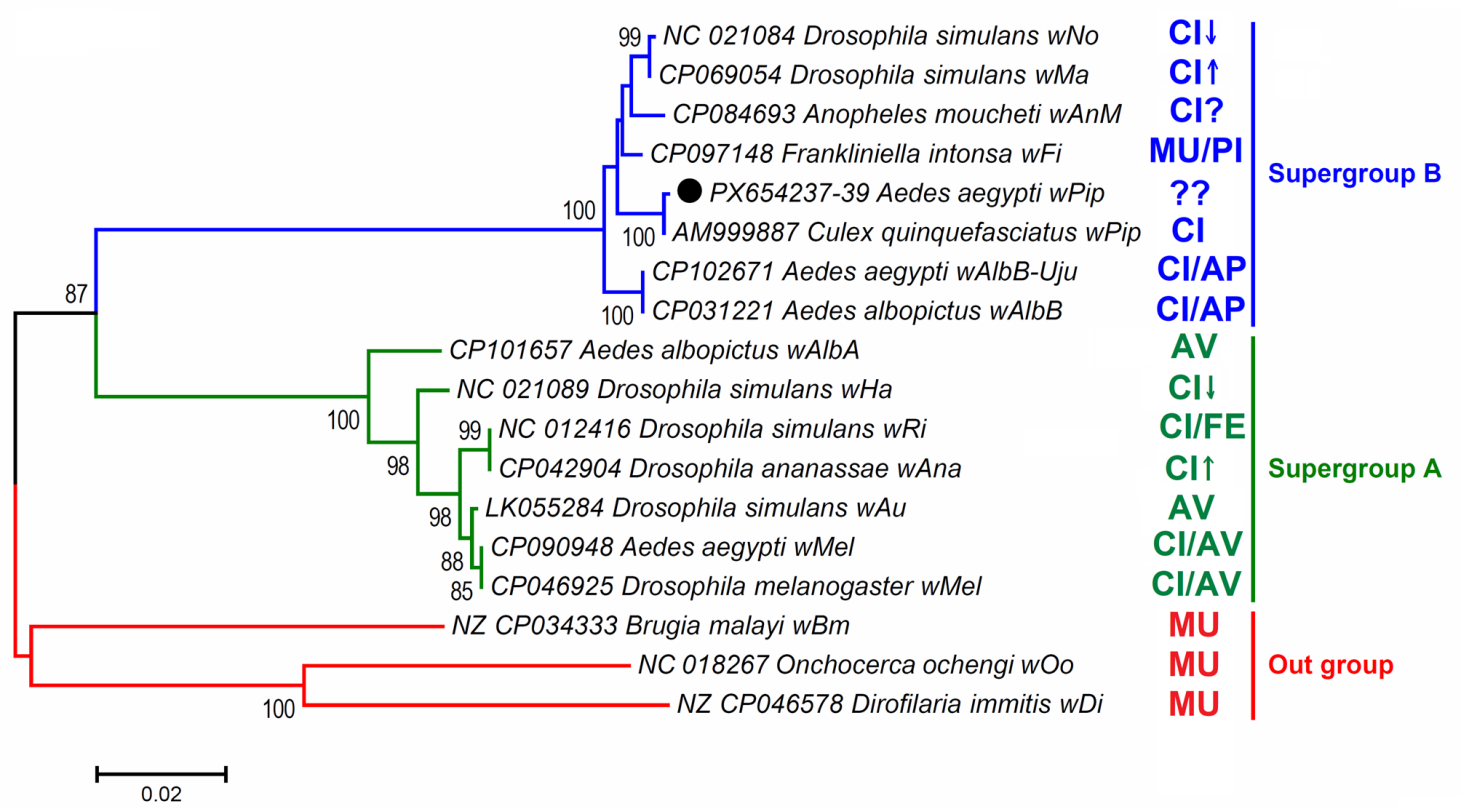
Maximum likelihood phylogenetic tree based on a 1405-bp fragment of the concatenated *Wolbachia ftsZ*, *gatB*, and *groEL* genes, illustrating the phylogenetic placement of *Wolbachia* strains isolated from *Aedes aegypti* (black circle) among related strains within supergroup A and B from other whole-genome sequenced *Wolbachia* specimens. *Wolbachia* sequences from *Brugia malayi* (NZ_CP034333), *Onchocerca ochengi* (NC_018267), and *Dirofilaria immitis* (NZ_CP046578) were used as outgroups to root the tree. Bootstrap support values (1,000 replicates) are shown at nodes; values < 50% are not displayed. The scale bar represents the number of nucleotide substitutions per site. Branch colors indicate *Wolbachia* supergroups: blue for supergroup B, green for supergroup A, and red for the outgroup. The right side of the tree indicates functional characteristics associated with each strain: CI: Cytoplasmic incompatibility, CI↑: Increased cytoplasmic incompatibility, CI↓: Reduced cytoplasmic incompatibility, MU: Mutualistic relationship, PI: Parthenogenesis induction, AV: Antiviral activity, AP: Antipathogen activity, and FE: Fitness effect.

MLST analysis of the three housekeeping genes (*ftsZ*, *gatB*, and *groEL*) confirmed the classification of *Wolbachia* strains obtained from *Aedes* mosquitoes in Iran. As shown in Fig. 7, the *Wolbachia* isolate from *Ae. aegypti* (PX654237-39) clustered with *wPip* strains from *C. quinquefasciatus* (AM999887) and formed a distinct lineage within supergroup B. This MLST-based phylogenetic analysis provides additional evidence for the presence of *wPip* in *Ae. aegypti* in Iran, confirming our initial *wsp*-based classification. The MLST profiles correspond to sequence types that have been previously associated with specific functional characteristics including cytoplasmic incompatibility patterns and pathogen blocking efficiency. Three MLST sequences generated in this study have been deposited in GenBank under accession numbers PX654237– PX654239.

## Discussion

Mosquitoes comprise ~ 3,600 species/subspecies in 41 genera globally, and 70 in 8 genera in Iran^22,29^— yet none rival the public health impact of invasive *Ae. aegypti* and *Ae. albopictus*. According to ECDC (early 2025), 640,349 dengue and 30,000 chikungunya cases—with 159 and 14 deaths respectively—were reported across 48 and 14 countries^30^. In Iran, syndromic surveillance over the past 11 months detected 1,126 dengue cases (921 endemic, 205 imported) and 5 imported chikungunya cases. This escalating burden underscores the urgent need for sustainable, eco-friendly interventions — such as *Wolbachia*-based strategies. Accordingly, this study assessed *Wolbachia* prevalence in lab- and field-collected *Ae. albopictus* and *Ae. aegypti* in Iran, finding an overall infection rate of 6%.

The low *Wolbachia* prevalence in the studied mosquitoes likely reflects the recent introduction of these invasive species to Iran — a pattern also observed in other newly colonized regions, suggesting early-stage establishment^31^. In contrast, longer-established populations often show higher infection rates, potentially due to natural transmission or human-mediated introductions^17,32^. This disparity is further supported here by higher infection rates in insectary-reared vs. field-collected specimens (Table 1). The low prevalence in *Ae. aegypti* may reflect transient infection or detection limits rather than stable colonization. While contamination is unlikely (given rigorous controls and replication), vertical transmission remains unconfirmed. Thus, the epidemiological significance of this finding should be considered preliminary, pending validation via FISH or maternal inheritance assays. These data may aid public health efforts to contain invasive mosquito spread.

In this study, the *Wolbachia* strains identified from *Ae. albopictus* and *Ae. aegypti* belonged to the *w*AlbB and *w*Pip subgroups, respectively. Both *w*AlbA and *w*AlbB strains have been reported in *Ae. albopictus*^33^; however, *w*AlbB demonstrates superior capabilities in pathogen blocking and reproductive manipulation in its natural host or when transfected into the corresponding vector^34,35^. In general, *Ae. aegypti* infected with *w*AlbB is less likely to spread infections of four serotypes of dengue virus via the salivary glands^36^. Additionally, the *w*Pip strain, which naturally infects the *Culex pipiens* complex and consists of five *w*Pip groups (*w*Pip-I to V) generates complex patterns of CI that enhance the effectiveness of *Wolbachia*-based suppression strategies for controlling *Ae. albopictus*^37–39^.

The MLST analysis provides robust confirmation of the strain identities initially determined through *wsp* sequencing. The *w*Pip strain detected in *Ae. aegypti* from Hormozgan Province (Fig. 7) represents a distinct lineage within supergroup B that has previously been documented in *Culex* species but rarely in *Aedes aegypti*. This finding is particularly significant as it confirms the natural occurrence of *w*Pip in *Ae. aegypti*, potentially representing one of the first documented cases globally. The MLST data further support our hypothesis that these strains may have been introduced through human-mediated transport rather than natural dispersal, given the genetic similarity to strains from distant geographic regions.

To our knowledge, this study is potentially the first report of natural infection of *Ae. aegypti* with the *w*Pip *Wolbachia* strain. The discovery of the *w*Pip strain in *Ae. aegypti* further confirms the ability of *Wolbachia* to overcome evolutionary, ecological, and immunological barriers. These adaptations enable *Wolbachia* to infect a wide range of hosts, manipulate their biology, and persist across generations^40^.

Studies have shown that *Ae. aegypti* mosquitoes carrying the *w*Pip, similar to those without *Wolbachia*, can still transmit dengue fever^41^. However, the same *Wolbachia* strain has been found to block transovarial transmission of Zika virus in *Ae. albopictus* mosquitoes^42^. The role of *w*Pip in enhancing the sterile insect technique has established it as an effective method or controlling *Ae. albopictus* populations^43^. Taken together, the release of *w*Pip-carrying *Ae. albopictus* and *w*AlbB-carrying *Ae. aegypti* may lead to the production of functionally sterile males and significant reductions in dengue transmission, respectively. Furthermore, by using strains such as ARwP-M, which combine *Wolbachia*-induced infertility and virus protection, it is possible to simultaneously target the reproductive biology and vector competence of a species. This approach results in a highly efficient and safe biocide for suppressing invasive mosquito populations^44^.

Just like its host mosquito, *Wolbachia* is sensitive to temperature changes and exhibits a positive temperature-dependent relationship^45^. This study highlights the discovery of the *Wolbachia w*AlbB isolates from *Ae. albopictus* mosquitoes adapted to cold climates, such as Ardabil, and the *w*Pip isolate from *Ae. aegypti*, which thrives in hot climates such as Bandar Abbas. Heat stress is known to have adverse, strain-specific effects on CI and *Wolbachia* load^46^. The *w*AlbB strain is relatively stable at high temperatures, which has facilitated its establishment in field trials in areas such as Kuala Lumpur, Malaysia. Consequently, *Ae. aegypti* mosquitoes carrying the *w*AlbB strain have been released as part of population suppression strategies that utilize CI to reduce the fertility of wild female mosquitoes^47^. The recent *Ae. aegypti* system carrying the *w*AlbB strain may function effectively in the extremely hot climate of Southern Iran, necessitating detailed studies and specific methodologies.

The potential climate sensitivity of *Wolbachia*–mosquito interactions highlights the necessity for adaptive, data-driven vector management strategies in Iran. Development of integrated surveillance platforms — combining entomological, microbiological, and meteorological data — is strongly recommended for public health and research institutions. Such systems are essential to anticipate and mitigate the impacts of climate variability on the efficacy of Wolbachia-based interventions, thereby ensuring their long-term resilience under projected climate change scenarios.

It is predicted that in the coming years, the spread of two invasive mosquito species in the mid-latitudes of the country will intersect, creating more challenges than the occurrence of each species individually. Factors such as mass gatherings and the movement of people within or outside the country during different seasons and annual ceremonies may facilitate the overlap of these two invasive mosquitoes^48^. The simultaneous presence of *Ae. aegypti* and *Ae. albopictus* has been reported in many regions worldwide^9^. Their coexistence poses significant public health challenges due to their roles as vectors for arboviruses. Effective surveillance, vector control, and public health interventions are essential to mitigate the risks associated with their overlapping distributions.

In this study, only a limited number of mosquito specimens tested positive in the *wsp* gene nest-1 PCR assay, with the majority of positive results observed in the nest-2 reaction (Fig. 3). This discrepancy likely reflects differences in *Wolbachia* density among the examined mosquitoes—a phenomenon previously documented elsewhere^49^. While our findings suggest the presence of *Wolbachia* in wild *Aedes* populations in Iran, they should be interpreted in light of certain methodological limitations. Specifically, we did not perform orthogonal validation using qPCR for bacterial density estimation, FISH for cellular localization, or direct assays for maternal transmission. Given the ongoing expansion of invasive *Aedes* mosquitoes in Iran and neighboring regions, future studies incorporating these complementary approaches will be essential to confirm *Wolbachia* infection status, characterize circulating strains, and assess their potential utility in biocontrol programs.

Such an integrated methodological framework, incorporating both *wsp* and MLST analyses, is essential to determine whether native *Wolbachia* infections—such as the *w*Pip strain detected herein—might enhance, interfere with, or otherwise compromise the efficacy of planned *Wolbachia*-based biocontrol strategies. The MLST data presented here provide a more comprehensive characterization of the strains circulating in Iranian *Aedes* populations, which will be critical for predicting potential interactions between native and introduced strains. The presence of *w*Pip in *Ae. aegypti* is particularly noteworthy as this strain has been shown to generate complex patterns of cytoplasmic incompatibility that could potentially interfere with the establishment of other *Wolbachia* strains used in biocontrol programs^37^^38^,.

Until a few years ago, wild *Ae. aegypti* mosquitoes, similar to *Anopheles* species, were not believed to be naturally infected with *Wolbachia*. However, recent studies have reported that the prevalence of *Wolbachia* is less than 5% in mosquitoes of American origin and approximately 25% in those of Southeast Asia. In New Mexico and India, this rate has exceeded 50%^50^. While the identification of natural *Wolbachia* infection in mosquitoes may represent a promising development in the control of invasive *Aedes* species, it could also pose a significant threat to the 16 countries implementing *Wolbachia*-based vector control strategies, potentially jeopardizing the success of these initiatives^51^.

## Conclusion

The global deployment of *Wolbachia*-based biocontrol strategies — exemplified by the World Mosquito Program and MosquitoMate — underscores the need for baseline data on native infections in target regions. This study documents low-prevalence natural infections of *w*AlbB in *Ae. albopictus* and *w*Pip in *Ae. aegypti* across Iran, with no detectable genetic heterogeneity among strains. While these findings represent a foundational step, they also highlight critical operational considerations: native *w*Pip in *Ae. aegypti* may interfere with CI-based interventions using introduced strains (e.g., *w*Mel, *w*AlbB), necessitating pre-release screening and long-term monitoring of strain interactions. In Iran — where both invasive *Aedes* species are expanding — integrating *Wolbachia* surveillance into vector control planning is not optional, but essential. Future large-scale studies employing diverse genetic markers are urgently needed to refine our understanding of *Wolbachia*–virus–mosquito dynamics and guide strain selection tailored to local epidemiological contexts.

## Supplementary Information

Below is the link to the electronic supplementary material.

Supplementary Material 1

## Data Availability

All relevant data are included within the manuscript. Sequence data have been deposited in GenBank under accession numbers PV122193–PV122209 and PX654237-PX654239. The datasets generated and/or analysed during the current study are available from the corresponding author, Naseh Maleki-Ravasan, on reasonable request.
