# Supplementary material for: Natural infection of Aedes albopictus with the wAlbB strain and Ae. aegypti with the wPip strain of Wolbachia in Iran
Source: Sci Rep. 2026 Feb 21;16:10070. doi: 10.1038/s41598-026-40993-7 (PMC13022244; doi:10.1038/s41598-026-40993-7)
Supplement: Supplementary file 1 — Supplementary Material 1 [file 41598_2026_40993_MOESM1_ESM.docx]

**Supplementary Table 1.** Geographic and climatic characteristics of mosquito collection sites in Iran.

| **Province** | **Location** | Coordinates (Lat., Lon.) | **Sampling Month** | **Mean Temperature (°C)** | **Mean Relative Humidity (%)** |
| --- | --- | --- | --- | --- | --- |
| Hormozgan | Bandar Abbas | 27.1963, 56.2884 | September | 32 | 75 |
|  |  |  | October | 28 | 65 |
| Ardabil | Bileh Savar | 39.3797, 48.3539 | July | 20 | 58 |
| Guilan | Bandar Anzali | 37.4639, 49.4799 | July | 22 | 85 |
|  |  |  | August | 23 | 86 |
|  | Khomam | 37.3911, 49.6596 | July | 23 | 83 |
|  |  |  | August | 24 | 84 |
|  | Shaft | 37.1702, 49.4019 | July | 23 | 83 |
|  |  |  | August | 24 | 84 |
| Mazandaran | Ramsar | 36.9268, 50.6431 | July | 27 | 70 |
|  |  |  | August | 28 | 72 |
|  | Tonekabon | 36.8154, 50.8711 | July | 27 | 71 |
|  |  |  | August | 28 | 73 |
| Insectary | Insectary | 35.74906, 51.06686 | January-December | 28 | 66 |

**Supplementary Table 2.** Logistic regression results for factors associated with species, site, and sex

| **Variable** | **OR** | **CI95%** | **p-value** |
| --- | --- | --- | --- |
| Species (Ae. aegypti vs albopictus) | 0.12 | 0.03–0.45 | 0.002 |
| Site (Guilan vs Insectary) | 0.28 | 0.10–0.75 | 0.01 |
| Site (Mazandaran vs Insectary( | 0.05 | 0.003–0.77 | 0.03 |
| Site (Hormozgan vs Insectary) | 0.14 | 0.03–0.62 | 0.009 |
| Sex (Female vs Male) | 0.88 | 0.42–1.85 | 0.74 |


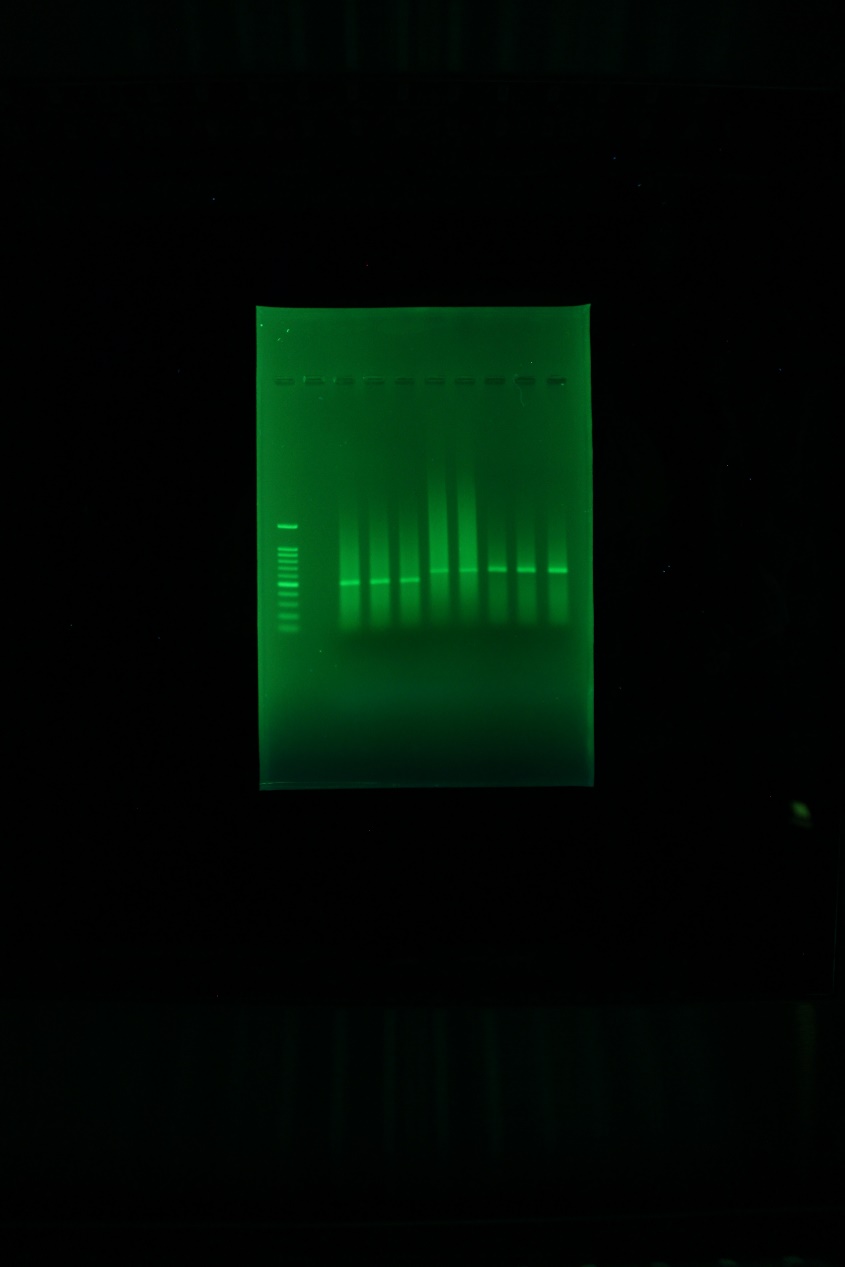


**Supplementary Figure 1.** Original unmodified gel image for Figure 3
